# Reply to Dr. E. K. Wedelstaedt

**Published:** 1904-03

**Authors:** M. L. Rhein

**Affiliations:** New York


### REPLY TO DR. E. K. WEDELSTAEDT.

BY M. L. RHEIN, M.D., D.D.S., NEW YORK.

In the January number of the International Dental Journal there appears an article by Dr. E. K. Wedelstaedt, which is intended as a reply to an article of mine in the September number, entitled "The Technique of Approximal Restorations with Gold in Posterior Teeth."

Dr. Wedelstaedt's article is so replete with errors, that it becomes a difficult matter to reply to the same and preserve the temperate demeanor which only is becoming to a scientific discussion. It is my intention to take up the points that he has raised, and answer them in order, as briefly as possible.

*First.* He starts in with a general objection to the praise and honor which was given to Marshall H. Webb in the article in question, but fails to reply to the only reason that Webb's name was introduced into the article. It was not my purpose to leave the impression that the method of preparing cavities pursued by Webb, or his method of inserting fillings, was distinctively new with him; but rather that it was a culmination of the practice of many men preceding and contemporary with him.

The main reason for bringing the name of Marshall H. Webb to the foreground in the article in question was to clearly demonstrate by the illustrations taken from his writings, and by his own words, that the principle of extending cavity margins for the prevention of recurrence of decay was well understood and practised by him. This was done because it had become a popular delusion in a certain section of the country that this was an entirely new doctrine evolved by Dr. G. V. Black. The only reply that Dr. Wedelstaedt makes to this point is, that they have stopped talking about extension for prevention in the Northwest.

It is rather a poor way of giving credit to a man who died practically a martyr to the cause of saving teeth. I say advisedly that he died a martyr to the cause, because the last few years of his active life were devoted almost exclusively to giving clinical demonstrations throughout the world, in the method of preparing cavities and properly filling them with gold. He did this at the expense of the necessary comforts for his own family, and in the course of this work contracted a cancer of the colon, which shortly after proved fatal.

In the models prepared by his own hand, and from which the drawings in his work were taken, there can be no question that Webb recognized the value of a flat gingival wall or seat. His students understood this thoroughly, and when Dr. Wedelstaedt says that "Dr. Rhein has at last recognized the value of having fillings properly seated on a flat gingival wall or seat," it is difficult to comprehend the necessity for such a statement, as this has been my practice for over twenty years. The same is true of occlusal anchorage in these cavities.

I regret that the illustrations of my article in the September number of this journal are done so poorly that it is difficult to understand them. This is not my fault. The blame is entirely at the door of the publishers, who made their cuts from actual models with natural teeth, which I turned over to them.

It was not intimated in my paper that Webb's preparation of cavities is precisely that of to-day. It is admitted that there have been important advances made, such as the elimination of retaining points; but the principles remain the same.

*Second.* The next point which Dr. Wedelstaedt takes up is his objection to finishing the cavo-surface angle with a sand-paper disk. In his well-known dogmatic style, he says, "Tight margins cannot under any circumstances be made against a smooth margin, if gold is the filling-material used." If Dr. Wedelstaedt had ever examined with a strong magnifying lens the ends of enamel-rods polished at their best, it is doubtful whether he would make such a statement. In reply, I would say that I consider it very objectionable to condense gold in the proper manner against the surface of enamel-rods which are necessarily left unsupported, if prepared by chisels, leaving a roughened surface such as he describes.

Furthermore it is denied that there is any tooth-surface at the occlusal portion so smooth that cohesive gold cannot be perfectly adapted to it, so as to make an hermetically sealed joint. His very statement indicates that he is unacquainted with the proper method of inserting cohesive gold so as to make a perfectly homogeneous plug. It might as well be stated here, that it is not possible to produce ideal results in cohesive gold by the use of pellets, cylinders, ropes, or any form except the regular foil or rolled gold. The use of any of these supposed labor-saving preparations, such as pellets or cylinders, is only productive of imperfect condensation and the leaving of air-spaces between the laminæ of the gold, and consequently preventing the accomplishment of what is most essential,—a homogeneous solid plug.

*Third.* The third and main objection which he finds to my article is the advocacy of an all-cohesive gold filling. The article in the September number was written distinctively for the purpose of bringing out discussion on this point. The cavities under consideration (approximo-occlusal in posterior teeth), when filled, must bear the stress of mastication, which we know varies from one hundred to three hundred pounds pressure.

If it can be proved that the fillings advocated by Dr. Wedelstaedt and his school, which consist of filling the gingival third with non-cohesive gold, will stand the same amount of stress as a perfectly condensed all-cohesive gold filling, there is no need of further argument. That Dr. Wedelstaedt claims such to be the case does not necessarily make it a fact, and part of my object has been to get at the real truth of this question. Dogmatic "say-so's" will not prove the correctness of this assertion on either side. In his article, he repeatedly refers to articles by Black published in 1891 and 1895 in the *Dental Cosmos,* in order to maintain his contention. A careful perusal of perhaps the most scientific articles on this subject that have ever been written—those just referred to—leaves in my mind only one impression. In such places as this it is impossible for us to obtain too great a density of our filling-material, in order to withstand the stress of years of wear.

In making his argument on this subject he mentions the fact that in the *Dental Cosmos* for 1895, commencing with page 749, there is given by Black a list of the specific gravity of different gold fillings. Three of these were made by Black himself. The remainder were made by different operators whose names are not given. The only ones in which a specific gravity above 19.3 was attained (which is the normal specific gravity of cast gold) were the three inserted by Professor Black himself. It appears from Dr. Wedelstaedt's article, that the three fillings inserted by means of the electro-magnetic mallet, and of which the highest specific gravity obtained was 13.2, were made by some one whom he calls an expert in the use of this mallet. From this he forms the conclusion that a filling made of cohesive gold, packed with the electro-magnetic mallet, is incapable of giving us the proper specific gravity.

In order to show that such a conclusion is incorrect, I made a filling out of the mouth under practically all the conditions set forth in my article, in the presence of two other dentists. The cavity was a distal approximate occlusal cavity of a superior molar. After the gingival half was completed, the filling was allowed to soak in water for an hour. The gold was then carefully smeared over with vaseline, so as to give it an opportunity to penetrate into the interstices, if possible. It was then carefully washed with chloroform and alcohol, and the surfaces freshened with a clean fissure bur. The filling was then continued precisely in the same manner as if this interruption had not taken place, and it was impossible, at the completion of the filling, and after the tooth had been cut in half and the filling removed, to tell where the point of union had taken place. Previous to its removal from the tooth, the filling had been polished in the ordinary manner. It was sent to the office of Dr. Ludwig Saarbach, a well-known expert, of this city, who gave as a certificate of its specific gravity, 19.87. When it is considered that this filling was inserted practically as one is placed in the mouth, so far as the amount of time and number of blows is concerned, the difference in the specific gravity obtained and that obtained by the so-called expert referred to by Dr. Wedelstaedt is worthy of notice.

It might be well to call attention to the fact that the highest specific gravity reported by Black was 19.42. If there should be any question as to the correctness of this specific gravity as compared with those stated in Black's article, there is one simple way of arriving at the truth,—and this has been tried before,—by an open competition of the various methods of packing gold.

At a clinic given before the First District Dental Society of the State of New York, in January, 1881, there was such a competition. It is peculiar that the simple expedient of obtaining the specific gravity of each filling was not thought of. Fillings were put in matrices of precisely the same mathematical dimension, and the following results were obtained:

By means of the electro-magnetic mallet, Dr. Webb inserted 9.65 grains.

With the automatic mallet, Dr. Ottolengui inserted 9.4 grains.

With the steel mallet, Dr. E. Parmley Brown inserted 9.25 grains.

With the lead mallet, Dr. Rynear inserted 9.2 grains.

With hand-pressure, Dr. Weld (who practically inaugurated the challenge) inserted 7.8 grains.

Only a similar form of experimentation, where the operators are known, can demonstrate the correctness or incorrectness of my assertion.

Dr. Wedelstaedt questions the ability of any operator to make tight margins with cohesive gold, especially at the gingival portion of a cavity. I might say, in reply, that the man who cannot perform such an operation, after a little practice, and having had the proper teaching, has but little claim to the confidence placed in him by patients who come to him for such work.

The frequent observation of my own fillings inserted in this manner, from the very commencement of my practice in 1881, and the fact that a large majority of them are doing as good service today as when they were inserted, is to my mind the strongest refutation of this argument. The whole matter is summed up in the fact that a great many dentists make a difficult operation out of the use of cohesive gold, because they have never been taught how easily it can be manipulated. Instead of being an "intractable material" as Dr. Wedelstaedt would have us believe, it becomes the most docile of agents when properly handled. It is on this account that my critic does not understand how a cohesive gold filling can be brought in a perfectly even surface from the gingival seat to the occlusal anchorage. It is on this account that he does not understand how a properly condensed filling of cohesive gold can be made in sections at different sittings, not only without any bad joint showing, but resulting in one homogeneous whole, and defying the detection of the joints.

Cohesive gold cannot be made into a solid homogeneous plug by means of the blow given by an instrument such as the automatic mallet, or a hand mallet, except at the expense of the most strenuous labor. It becomes, however, one of the easiest of operations under the direction of the electro-magnetic mallet with a current directed from a battery, so that the instrument is capable of giving at least three thousand five hundred blows per minute. By this means, the pieces of gold-foil laid upon the portion of the filling already inserted are not punched into the filling already made, but are carefully ironed into the filling through the aid of the innumerable blows given. It is my contention that with this form of manipulation the highest specific gravity of gold can be attained. It must not be overlooked that in the proper manipulation of cohesive gold a most essential feature is to prevent the gold coming in contact at any time with any foreign substance except carefully polished instruments and the surface of the annealer. Any deviation from this rule will imperil the perfection of the filling and is too frequently the sole cause of failure.

My critic goes to considerable length in taking exception to the plan which I advocated for anchoring my first piece of cohesive gold. He states that in examining three such fillings which have been anchored by this method, he found that the cement used was simply a white powder. He does not say what methods he has used in order to make his examination. I might reply that, in the course of similar examinations, I have found a condition precisely similar to the removal of a porcelain inlay after it has been set,—viz., a portion of the cement adhering to the inner periphery of the cavity, and another portion adhering to the surface of the gold. In other words, that it was impossible to remove the cement in a clean way from either the gold or the tooth surface, which I consider the ideal result to be obtained. I do not deny that this cement is capable of being reduced to a powder similar to the condition it was in before it was made into a cement, but I have never seen it in this pulverized state by simply dislodging the filling from the broken tooth. It might be well to state that the anchorage of a gold filling in this manner produces all the advantages that are claimed from a porcelain inlay that has been cemented into place.

Dr. Wedelstaedt has laid considerable stress in his article on the advantage of measuring the size of his tooth and the cavity in its different relations. He then proceeds to estimate how much non-cohesive gold such an approximate surface will stand and still retain sufficient solidity, from the amount of cohesive gold on the occlusal portion, to withstand the stress of ordinary use. It has never yet been my good fortune to meet with one operator, whose work I have valued, who can find any practical advantage from the system of cavity measurements which has been so extensively taught by Dr. Wedelstaedt.

It appears to me that this millimetre equation is fit to be placed in the same category with the problem of, "Given the age of Mary, how old is Ann?" It is impossible to teach the insertion of gold fillings in these surfaces by any mathematical calculation such as is used in building operations, for the simple reason that we never find two mouths in which exactly similar conditions prevail. If the majority of teeth that presented themselves to us conformed even in an ordinary ratio to the form which we designate as the correct physiological one, the filling of teeth might logically be taught in such a manner. As it is, however, the exceptions to the normal position of teeth are the rule, and it becomes the problem of the dentist in this form of cavity, how to best give to those teeth the most serviceable form of contact point, and one which will best preserve the health of the interproximal space at the gingival angle.

In many of these cases the operator is compelled to perform operations that must be devised entirely for the case in hand, and it is absolutely impossible, under such circumstances, to rely upon any of Wedelstaedt's equations.

In another place Dr. Wedelstaedt speaks of the fact of his seeing failures of fillings made with all-cohesive gold. I might say, with perfect candor, that I have seen the same thing in all-cohesive gold fillings, and at the same time a great many more failures where non-cohesive gold and gold-and-tin fillings have been used in the gingival third. I need not call the attention of any operator who has removed even a single non-cohesive gold filling to the vile odors which emanate from the separated laminæ of the gold which burs out like wet sand.

This sort of observation will not prove that either method of filling teeth cannot be used so that the teeth can be saved permanently. The only question at issue is, Which method properly pursued will best preserve the teeth? In this respect, I might call attention to the fact that if the gold is more solidly condensed, if it is used in a cohesive manner at the gingival third,—supposing that it is correctly placed in position,—it certainly will be better able to withstand the scaling of those places in the years following a supposed good operation by either method.

I have seen fillings, where the gingival third was filled with non-cohesive foil, that have been practically ruined in the scaling of the teeth by the careless instrumentation of the operator.

There are a number of places in Dr. Wedelstaedt's article in which he simply says that the operation advocated in my article cannot be done, and consequently cannot be endorsed. There is nothing to be gained in this kind of talk, nor in answering a criticism like this. If, however, in open contest my critic can demonstrate the falsity of my views, or the superiority of those which he so grandiloquently maintains, I shall be only too pleased to change my venerable form of practice for one that is capable of giving more benefit to my patients.

